# Anhedonia Behavioural Activation and Ketamine Therapy (AAKT): Shaping a Proposal With Patient and Public Involvement

**DOI:** 10.1192/bjo.2025.10791

**Published:** 2025-06-20

**Authors:** Anya Borissova, Allan Young, Mitul Mehta, Tim Dalgleish, Camilla Nord

**Affiliations:** 1 South London and Maudsley NHS Foundation Trust, London, United Kingdom; 2 King’s College London, London, United Kingdom; 3 Imperial College London, London, United Kingdom; 4 University of Cambridge, Cambridge, United Kingdom and Et al.: Dr Anna Bevan and Dr Liliana Galindo (University of Cambridge)

## Abstract

**Aims:** Ketamine and behavioural activation (BA) reduce levels of anhedonia in major depressive disorder (MDD), though neither resolves anhedonia. Combining these treatments could yield additive or even multiplicative benefits.

This poster summarises the development of a PhD fellowship proposal, discussing: (1) the initial patient and public involvement (PPI) interviews, exploring views on participation in ketamine research; (2) the resulting proposal, assessing the feasibility of a trial augmenting BA with IV ketamine for MDD and anhedonia.

**Methods:** 1. One-to-one PPI interviews were conducted with seven participants from a previous study of ketamine in treatment-resistant depression. Questions addressed (a) volunteering reasons, (b) positives to retain and areas to improve and (c) research objectives opinions.

2. Participants with MDD and anhedonia will be recruited from the community and local primary care services (GP and NHS Talking Therapies). A planned feasibility parallel-group randomised controlled trial will compare IV ketamine with BA (n=20), against midazolam with BA (n=20). Participants will attend eight visits (including one-month follow-up) over 10–12 weeks, receiving 3 ketamine or midazolam infusions in between 6 BA sessions. Feasibility of study procedures and collection of outcome measures will be assessed.

**Results:** A key motivator for participants to volunteer was hope that ketamine would improve depression symptoms. Some were curious about ketamine’s effects. They wished to support research into depression treatments.

Most participants experienced a stark difference between ketamine and midazolam (placebo). They described feeling more open, malleable and involved in the world after ketamine.

Participants suggested future studies retain a focus on treating participants as individuals rather than a ‘number’ and allowing unrushed sessions. They thought clinician-led interviews helped reflect on symptom changes.

Participants found the consent process thorough. However, some suggested it should be clearer that mood changes from ketamine may not last. They recommended providing audiovisual materials on the ketamine experience/infusion room to support preparation. They suggested a check-in between infusion sessions and a final session to discuss onward referrals, medication advice and signposting to low-cost counselling and integration groups.

Most thought ketamine could enable better use of therapy time and that therapy could help make sense of thinking changes post-ketamine. Other suggestions for research included duration of symptom relief post-ketamine, treatment accessibility including in primary care, and medication side effects.

**Conclusion:** PPI interviews supported combining ketamine with psychotherapy. They provided key insights on improving study procedures to support research into augmenting primary care therapies for depression treatment.

